# Bismuth organic frameworks exhibiting enhanced phosphorescence

**DOI:** 10.1038/s42004-021-00607-x

**Published:** 2021-12-02

**Authors:** Jin Young Koo, Changmin Lee, Taiha Joo, Hee Cheul Choi

**Affiliations:** Department of Chemistry, Pohang University of Science and Technology (POSTECH), 77 Cheongam-ro, Nam-Gu, Pohang 37673 Korea

**Keywords:** Metal-organic frameworks, Optical materials

## Abstract

Bismuth-based organic frameworks (BiOFs) can display interesting phosphorescent properties, but the relationship between structure and optical activity remains underexplored. The structure-dependent phosphorescence properties in the BiOFs are investigated using different multidentate ligands. In-depth analysis of the luminescence properties confirms that the densely packed framework shows long-lasting phosphorescence at room temperature, owing to an efficient electron-hole separation. The combination of spectroscopic analysis and single-crystal structural analysis provides important insights into the emission control through BiOFs structural change, which can be a useful strategy for modulating the optical properties of various metal organic frameworks. Furthermore, taking the advantage of long-lasting phosphorescence, the potential usage as an eco-friendly photocatalyst is demonstrated.

## Abstract (web summary)

Bismuth organic frameworks can display interesting phosphorescent properties, but the relationship between structure and optical activity remains underexplored. Here two bismuth organic frameworks with differing bismuth coordination number and intermetallic distance are contrasted.

## Introduction

Bismuth is an attractive metal ion for optoelectronic applications because of its high atomic mass leading to high spin–orbit coupling (SOC), resulting in interesting optical properties when formed in organobismuth complexes^1–4^. Among them, bismuth-based phosphorescent MOFs are highly attractive for high potential applications such as organic light-emitting diodes for display or bioimaging^5–8^. Bismuth has low toxicity, relatively low cost and good chemical stability compared with the commonly used heavy metals. Despite these advantages, phosphorescent properties derived from pure bismuth-based MOFs are rarely reported. While we are searching organic ligands that can chelate to Bi and contribute to the optical property of Bi, we have found a seminal work by Feyand et al.^9^, describing bismuth-based organic frameworks (BiOFs) including Bi-pyromellitic acid complex (Bi-BTCA). Although this work dose not discuss the phosphorescence property of the BiOFs in detail, since their complex is an important system to explore the structure-luminescence property of Bi complexes by controlling the structure using diverse organic ligands, we revisit Bi-BTCA crystal, especially focusing on its phosphorescence property changes by comparing it with Bi-trimesic acid complex (Bi-BTC) crystal that offers different coordination number of Bi and interbismuth distance, because the luminescence of bismuth complexes is highly sensitive to their structure parameters such as interbismuth distance, etc. For example, we have recently reported photoluminescence (PL) property from triphenyl bismuth complexes formed through noncovalent interactions among triphenyl bismuth molecules^10^.

Herein, we report different phosphorescence properties from Bi-BTCA and Bi-BTC crystals, which mainly owe to the different interbismuth distances in both crystals. The structure-luminescence correlated property has been studied using X-ray analysis and steady-state PL and time-resolved PL (TRPL) measurements. In addition, due to the long phosphorescence of Bi-BTCA, it exhibits effective photocatalytic activity, as confirmed by the degradation of dye molecules under visible light irradiation. Our study shows not only that BiOFs can be highly luminescent materials with a careful control of its structure but also will extends the field of an eco-friendly catalyst beyond the precious metal-based MOF catalyst.

## Results and discussion

### BiOF with tetracarboxylates ligand (Bi-BTCA)

Bi-BTCA is prepared by the conventional solvothermal method using bismuth iodide (BiI_3_) and pyromellitic acid (H_4_BTCA) in distilled water at 180 °C for 72 h. The scanning electron microscopy (SEM) and optical microscope (OM) measurements show that the crystals are sucessfully formed with high homogeniety in shape (Supplementary Fig. 1). They are colorless rod shape crystals at micrometer-scale length with a flat surface.

**Fig. 1:**
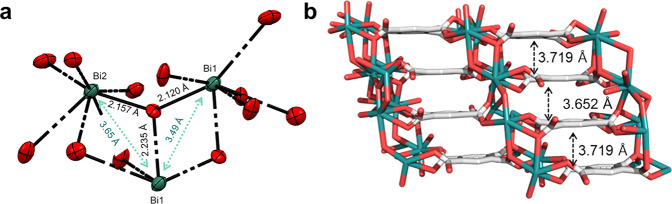
Crystal structure of Bi-BTCA. **a** ORTEP view of the bismuth coordination geometry with 50% thermal ellipsoids. **b** Network having a 3D parallel packing structure along the *b* direction with a face-to-face stacked orientation. Although hydrogen atoms are omitted here for clarity, they were considered in the calculation of the accessible volumes. Green, bismuth; gray, carbon; red, oxygen.

The infinite three-dimensional coordination framework has a triclinic (*P*-1) space group, which agrees well with the previous report^9^. Two notable features of Bi-BTCA are (1) the shortest interbismuth distance is 3.49 Å, which is close enough to induce strong interaction (Fig. 1a) and has shorter Bi–O bond distances, between 2.1 and 2.2 Å compared to the previously published bismuth-based carboxylate MOFs (typically in the range of 2.4–2.8 Å)^11^. (2) Due to the strong interaction between ligand and metal, the planar BTCA molecules are well-arranged along the *b*-axis direction and show a slipped face-to-face stack orientation with a distance of shorter than 3.8 Å with no void space (Fig. 1b) (Supplementary Data 1). In addition, the powder X-ray diffraction pattern of the as-prepared Bi-BTCA is well matched with the simulated pattern obtained from the Mercury 3.0 software (Supplementary Fig. 2). The limited number of diffraction peak indicates that the Bi-BTCA crystals have a preferred orientation^12,13^. This result clearly indicates that the obtained Bi-BTCA has a pure crystalline phase. The thermal stability of Bi-BTCA has been examined by thermogravimetric analysis (TGA), revealing that the crystal collapses at temperature beyond 731 K (onset temperature) (Supplementary Fig. 3). This result indicates that the Bi-BTCA structure induced by multiple bonds between Bi and ligands has significantly high thermal stability.

**Fig. 2:**
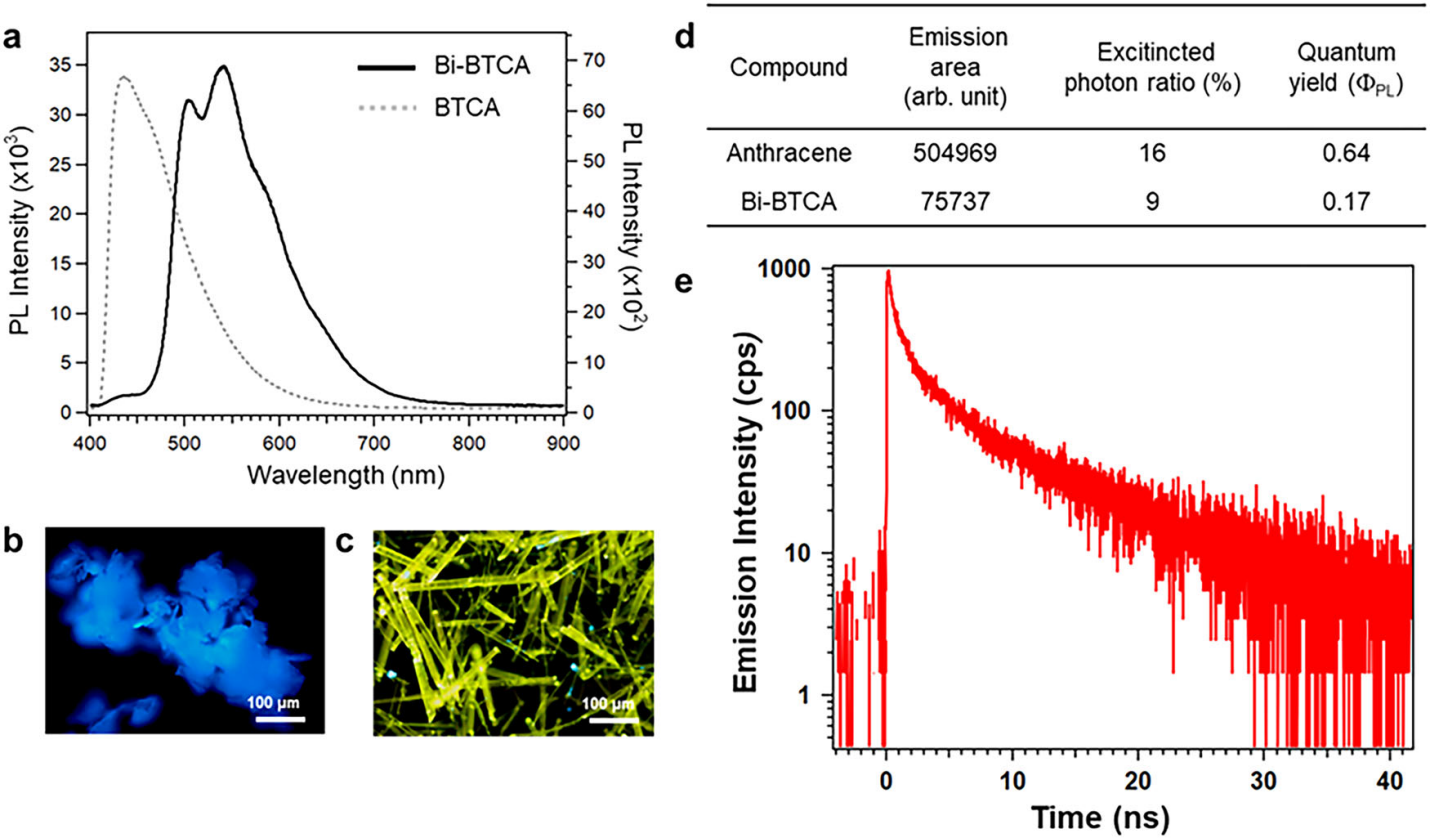
Photophysical characterization of Bi-BTCA. **a** Steady-state PL spectra of BTCA (dotted line, left axis) and Bi-BTCA crystal (solid line, right axis). **b** BTCA and **c** Bi-BTCA crystals optical images taken during exposure to a UV (365 nm) lamp. **d** Photoluminescence quantum yields obtained in single crystals. **e** TRPL of Bi-BTCA (red) crystal detected at 500 nm after photoexcitation at 380 nm. Time resolution was 75 ps.

A large SOC of bismuth can lead to long-lived triplet excited states through intersystem crossing, which is expected to facilitate charge separation. Therefore, the steady-state PL spectrum of Bi-BTCA is measured to investigate its photophysical properties. The spectrum shows its optical property with a very strong emission with a maximum at 540 nm and a narrow full width at half maximum of 126 nm (0.42 eV). The spectrum is red-shifted from the pure ligand that shows emission at 430–470 nm (π→π* or n→π* transition)^14^ (Fig. 2a). According to the CIE chromaticity diagram, Bi-BTCA shows the coordinates of 0.34 and 0.48, which can be assigned as yellow light, which also matches well with the OM image (Supplementary Fig. 4, Fig. 2b, c). The PL quantum yield (PLQY, Φ_PL_) of Bi-BTCA crystals has been measured with a reference of anthracene crystals grown by sublimation^15^. The Φ_PL_ for Bi-BTCA in crystalline phase is 0.17 (Fig. 2d).

To elucidate the origins of PLQYs, additional TRPL experiments are conducted. To record the TRPL, a time-correlated single photon counting method is used. Detailed procedure is described in the “Method” section. Figure 2e shows the TRPL of Bi-BTCA (red) excited at 380 nm and detected at 500 nm, and the time resolution is 75 ps. The measured TRPL is fitted to the exponential functions. The decay time constants of Bi-BTCA are 470 ps (61%), 2.4 ns (27%), 12 ns (10%), and 540 μs (1.4%), which give the average lifetime of 540 μs. Long time component of TRPL of Bi-BTCA is shown in Supplementary Fig. 5. Bi-BTCA has not only long decay time, but more importantly, it has long-lasting PL with 540 μs of time constant, which has not been known before.

A long lifetime of the excited state of Bi-BTCA means the photogenerated charge carriers can live long before they decay back to the ground state, leading to more efficient electron–hole separation, which can be utilized for photocatalysis. The photocatalytic activity of Bi-BTCA is examined for the degradation of methylene blue (MB) and rhodamine B (RhB), which are the most abundant dyes in the textile industries effluents and commonly chosen as a model pollutant for the photocatalytic efficiency test (Supplementary Fig. 6). As a result, Bi-BTCA having long-lasting PL exhibits successful degradation of RhB (96.46%) and MB (98.51%) dye molecules under visible light irradiation, whereas the degradation of MB was negligible in the dark.

### BiOF with tricarboxylates ligand (Bi-BTC)

To investigate the effect of the structure (especially interbismuth distance) on long-lasting phosphorescent Bi-BTCA, Bi-BTC crystals using a tricaboxylate ligand (H_3_BTC) that has three carboxyl groups are synthesized to make a crystal having an increased interbismuth distance. The H_3_BTC ligand is expected to keep the interbismuth distance longer by aparting the carboxylate groups to which bismuth ions bind. Bi-BTC network crystals are successfully synthesized by the conventional solvothermal method using BiI_3_ and H_3_BTC in a mixture of dimethylformamide (DMF) and toluene. The SEM and OM images show that all resulting crystals are thin and colorless having a length at micrometer-scale with a flat surface (Supplementary Fig. 7).

**Fig. 3:**
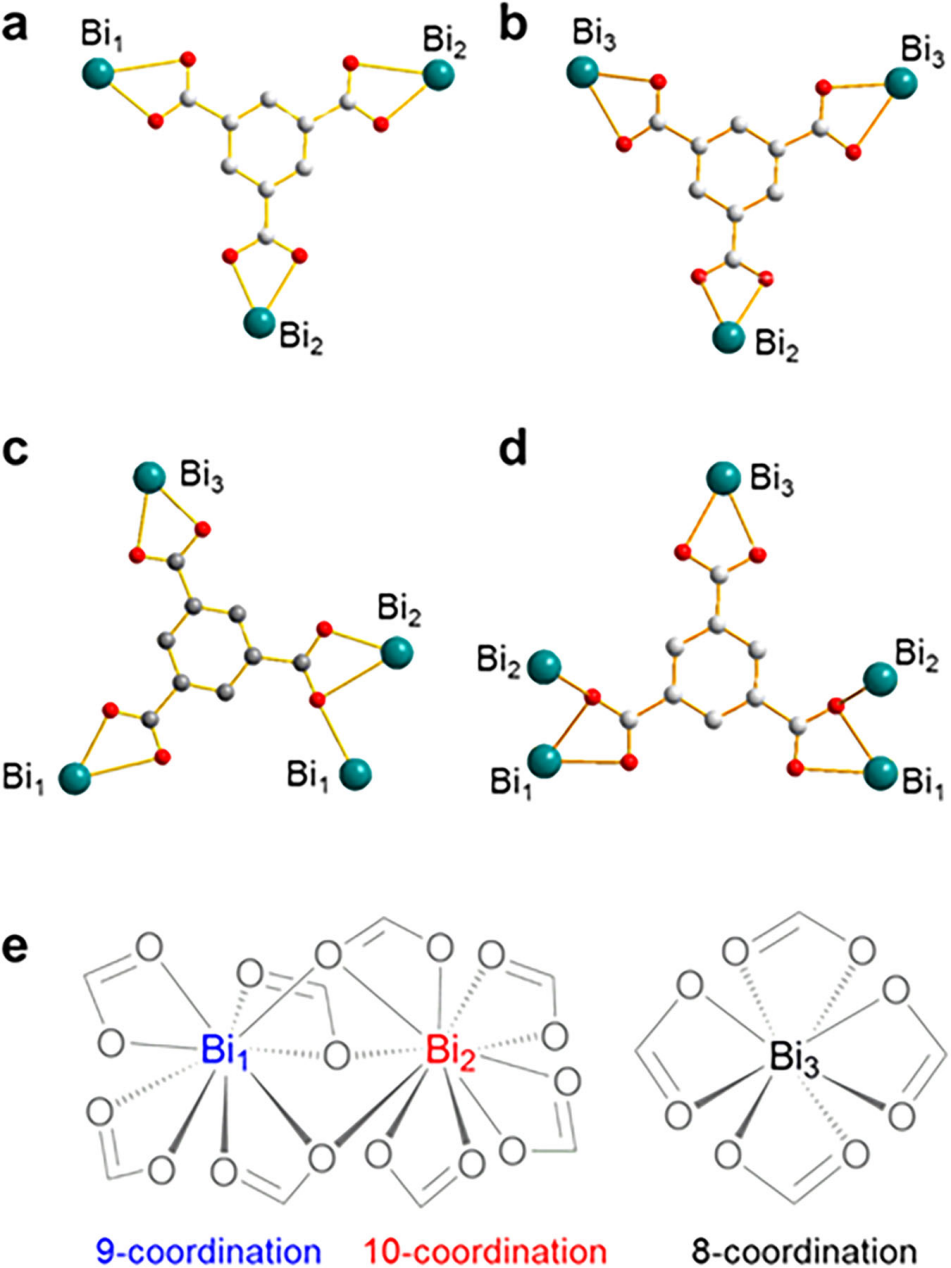
Coordination mode of Bi-BTC. **a**–**d** Coordination geometry of BTC ligand bound to each Bi^3+^ center in four different manners. **e** Three crystallographically independent Bi^3+^ metal centers; binuclear {Bi_1_-Bi_2_} centers are bridged by three carboxylate ligands.

Unlike Bi-BTCA, Bi-BTC has a complex structure with a trigonal prismatic network. The key structures of Bi-BTC are shown in Fig. 3, showing an asymmetric unit of Bi-BTC built from three crystallographically independent Bi^3+^ metal centers, and four different coordination geometries of BTC ligand (Fig. 3a–d). Each Bi^3+^ metal center is bound to O atoms from carboxylate group of ligands and in particular, binuclear {Bi_1_-Bi_2_} centers are bridged by three carboxylate ligands. A remarkable feature of Bi-BTC is that each Bi^3+^ ion has three different coordination numbers from 8 to 10, leading to a complex structure granting a high porosity (Fig. 3e) (Supplementary Data 2). To the best of our knowledge, this is the first report of bismuth ions at 8, 9 and 10 coordination numbers coexisting in one system. In addition, all carboxylate sites in BTC are presumed to be deprotonated like BTCA, and four anionic carboxylate groups are chelated to Bi^3+^, resulting in a negatively charged framework that is balanced by dimethylammonium cations which are generated in situ upon solvent decomposition during the solvothermal synthesis. Furthermore, the network exhibits Bi–O bonding distances between 2.4 and 2.8 Å, which are longer than Bi-BTCA. Thus, unlike Bi-BTCA that has several adjacent bismuth ions, Bi-BTC has two adjacent bismuth ions with a distance of 3.92 and 4.03 Å, which is notably longer than the one in Bi-BTCA (Supplementary Fig. 8). Furthermore, owing to the complex coordination geometry, the structure has an effective contact surface of ~44.8% (2677.08 Å) per unit cell volume as calculated by using Mercury 3.0 after removal of guest and water molecules (Supplementary Fig. 9). In this regard, it is also found that three independent pores are present in the network including two trigonal pores (yellow and blue), and one independent rectangular pore (green) with accessible diameters of 4.0, 4.0, and 3.1 Å, respectively, as calculated accounting for the van der Waals radii of carbon and oxygen atoms of the pore walls (Supplementary Fig. 10a, b). All channels are aligned parallel to the *c*-axis and the pore morphologies (indicated by yellow surfaces) show a honeycomb-shaped channel surface which can highly interact with guest molecules (Supplementary Fig. 10c). To know the structural stability, TGA is performed. TG curves of Bi-BTC show weight losses corresponding to solvent removal at 300–339 and 339–452 K and decomposition at above 452 K (onset temperature is 628 K) (Supplementary Fig. 11), which is 100 K lower than Bi-BTCA. This result indicates that Bi-BTC is less thermal stable than Bi-BTCA. In addition, the possible existence of iodide residue of both BiOFs has been examined by measuring the energy-dispersive X-ray spectrum (Supplementary Fig. 12). The result clearly indicates that the crystals are free of iodide residues.

**Fig. 4:**
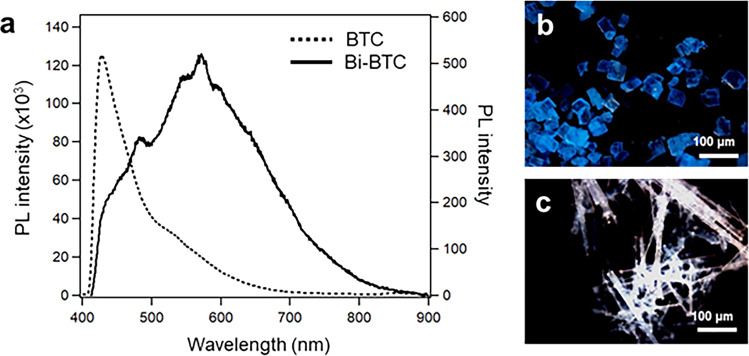
Photophysical characterization of Bi-BTC. **a** Steady-state PL spectra of BTC (dotted line, left axis) and Bi-BTC crystal (solid line, right axis). **b** BTC and **c** Bi-BTC crystals image taken during exposure to a UV (365 nm) lamp.

The effect of interbismuth distance on the luminescence property of Bi complexes is elucidated by comparing the PL property of Bi-BTC crystals having a longer interbismuth distance than Bi-BTCA. The steady-state PL spectra of Bi-BTC crystals show a totally different emission property compared to Bi-BTCA crystals (Fig. 4). Bi-BTC crystals show a weak and broad emission bands, with a maximum emission at 590 nm and a large full width at half maximum of 219 nm (0.88 eV) (Fig. 4a). Compared with BTC ligand itself that shows an intense emission at 410 nm (π*→π transition)^8^, Bi-BTC shows a red-shift and broadening tendency with a reduced intensity. Figure 4b, c shows OM of BTC and Bi-BTC crystals under UV (365 nm) irradiation, respectively. In addition, TRPL experiment confirms that Bi-BTC exhibits relatively fast electron–hole recombination. Quantum yields are measured by steady-state spectra with the quantum yield reference crystal anthracene. As a result, quantum yield (Supplementary Fig. 13) of Bi-BTC is certainly smaller than Bi-BTCA. Bi-BTC decays with time constants of 160 ps (60%), 1.2 ns (34%), and 5.8 ns (9%), which gives an average lifetime of 3.5 ns. These results indicate that there is a nonradiative decay channel in Bi-BTC that makes the long-lasting exciton decays much faster than in Bi-BTCA. Their short lifetime is well matched with the low photocatalytic activity of Bi-BTC (Supplementary Fig. 14). The degradation of MB was negligible in the dark and only about 10% of MB was degraded for 160 min visible light irradiation in the presence of Bi-BTC.

Meanwhile, the needle-shaped Bi-BTC crystals show pure white light emission upon UV irradiation. To clearly compare the white light PL emission of Bi-BTC to the standard white light, their CIE coordinates are determined according to CIE 1931 chromaticity based on its PL spectrum. The PL emission from Bi-BTC shows the coordinates of 0.34 and 0.33 in the CIE chromaticity diagram, which are close to the pure white light (0.33, 0.33) (Supplementary Fig. 15). These results indicate that a bismuth-based network can be applied as an effective white light-emitting material without any supporting material. This is very meaningful considering that most of the white emitting MOFs that are available in the literature are based on rare earth metal doping or the encapsulation of some luminescent dye molecules or other inorganic complexes within the pores of MOF^16,17^.

## Conclusion

The effect of interbismuth distance in a bismuth complex on its luminescence property was investigated using different chelating ligands. While Bi-BTCA having a shorter interbismuth distance exhibits a long-lasting phosphorescence, Bi-BTC having a longer interbismuth distance exhibits weak phosphorescence. In addition, Bi-BTCA shows an effective photocatalytic activity as confirmed by the degradation of dye molecules under visible light irradiation. Owing to the advantages of the visible light response, stable structure, and low cost, this bismuth-based photocatalyst will boost the development of eco-friendly catalysts beyond the precious metal-based MOF catalysts.

## Method

### Synthesis

#### Bi-BTC

BiI_3_ (58.0 mg, 0.1 mmol) and H_3_BTC (63 mg, 0.3 mmol) were dissolved in 1:2 mixture of DMF/toluene (6 mL). The reaction mixture was added into a 20 mL teflon vessel and sealed using a high temperature hydrothermal reactor and placed in a furnace at 130 °C for 72 h.

#### Bi-BTCA

BiI_3_ (58.0 mg, 0.1 mmol) and H_4_BTCA (76.2 mg, 0.3 mmol) were dissolved in DI water (10 mL). The reaction mixture was added into a 20 mL teflon vessel and sealed using a high temperature hydrothermal reactor and placed in a furnace at 180 °C for 72 h.

#### Anthracene crystal

The physical vapor transport process was performed by placing 10 mg of anthracene powder (Aldrich, Reagent Plus^®^ 99%, melting point (Tm) = 210–215 °C) at the center of a tube-type furnace with a product-collecting Si substrate placed at the downstream end of the furnace. The vaporization temperature measured at the location of anthracene powder was 200 °C. The temperature at a Si substrate was 45 °C, and the growth proceeded for 12 min after the furnace reached at 200 °C (flow rate of carrier Ar = 200 sccm) produced colorless platy crystals. The product was used without further purification. 1H NMR (500 MHz, CH3CN-d5) δ 8.096 (q, 4H, J = 3.5 Hz), 7.538 (q, 4H, J = 3.5 Hz), 8.551 (s, 2H).

### Single-crystal X-ray diffraction structure characterization

All Bi-BTC and Bi-BTCA crystals to examine were coated with paratone-N oil and the diffraction data were measured at 100 K with a synchrotron radiation (*λ* = 0.60997 Å) and at 219 K with a synchrotron radiation (*λ* = 0.61000 Å), respectively, using ADSC Quantum-210 detector at BL2D SMC with a silicon (111) double crystal monochromator (DCM). The ADSC Q210 ADX software^1^ was used for data collection and HKL3000sm (version 715)^2^ for cell refinement, reduction. Absorption correction was performed by using the program PLATON^3^. All crystal structures were solved by direct method and refined by full-matrix least-squares calculation using SHELXL-2017 programs^4^. All the nonhydrogen atoms were refined anisotropically. All hydrogen atoms were added to their geometrically ideal positions. During the Bi-BTC refinement, we used four OMIT instructions to remove the number of reflections for which I(obs) and I(calc) differ more than ten times sigmaW.

#### Bi-BTC [Bi_6_(C_9_O_6_H_3_)_8_(DMA)_10_(H_2_O)_10_]

C_92_H_24_O_58_N_10_Bi_6_, *M*r = 3451.09, crystal dimensions 0.1 × 0.02 × 0.01 mm^3^, orthorhombic Pnnm, *a* = 21.732(4) Å, *b* = 28.072(6) Å, *c* = 9.885(2) *α*, *β*, *γ* = 90°, *V* = 6060(2) *T* = −173 °C, *Z* = 2, *ρ*_calcd_ = 1.899 g cm^−3^, μ = 5.945 mm^−1^, 8623 unique reflections out of 14292 with *I* > 2*σ* (*I*), 455 parameters, 1.60° < *θ* < 29.88°, *R*_1_ = 0.0531, *wR*_2_ = 0.1384, GOF = 0.958.

#### Bi-BTCA [Bi_2_(C_10_H_2_O_8_)·H_2_O]

C_10_H_2_O_9_Bi_2_, *M*r = 684.08, crystal dimensions 0.17 × 0.02 × 0.02 mm^3^, triclinic *P-1*, *a* = 7.8670(16) Å, *b* = 8.4420(17) Å, *c* = 9.890(2) Å, *α* = 108.92(3)°, *β* = 90.80(3)°, *γ* = 116.32(3)°, *V* = 547.2(4) Å^3^, *T* = −56 °C, *Z* = 2, *ρ*_calcd_ = 4.152 g cm^−3^, μ = 21.678 mm^−1^, 2646 unique reflections out of 5343 with *I* > 2*σ* (*I*), 192 parameters, 1.90° < *θ* < 33.02°, *R*_1_ = 0.0762, *wR*_2_ = 0.1867, GOF = 0.967.

### Photocatalytic experiment

The photocatalytic activity of both BiOF crystals was evaluated using MB. The LDLS (EQ-99FC) with UV-cutoff filter was used to provide visible light with *λ* ≥ 410 nm and the absorbance of MB was monitored using a UV/visible spectrophotometer (UV-2600, Shimadzu). The visible light source was placed in the middle of the quartz vessel and set about 10 cm from the sample. The reaction was initiated by switching on the light source after adding the composite to dye solutions.

The dye degradation efficiency was measured by the following:

1 $${{{{{{{\mathrm{Degradation}}}}}}}}\left( \% \right) = \left[ {\left( {{{{{\mathrm{C}}}}}_0 - {{{{\mathrm{C}}}}}} \right){{{{{{{\mathrm{/}}}}}}}}{{{{\mathrm{C}}}}}_0} \right] \times 100$$

where C_0_ is the concentration of the dye in water before irradiation and C is the concentration of the dye in water after a certain irradiation time.

## Supplementary information

Description of Additional Supplementary Files

Supplementary Information

Supplementary Data 2

Supplementary Data 1

## Data Availability

All data and characterization of this study are included in this article and its Supplementary Information. The X-ray crystallographic coordinates for structures reported in this article have been deposited at the Cambridge Crystallographic Data Centre (CCDC), under deposition number CCDC 1976709, contains the supplementary crystallographic data for Bi-BTC, and CCDC 2005083, contains the supplementary crystallographic data for Bi-BTCA. These data can be obtained free of charge from the CCDC via www.ccdc.cam.ac.uk/data_request/cif.
