# Supplementary material for: Bismuth organic frameworks exhibiting enhanced phosphorescence
Source: Commun Chem. 2021 Dec 2;4:167. doi: 10.1038/s42004-021-00607-x (PMC9814738; doi:10.1038/s42004-021-00607-x)
Supplement: Supplementary file 1 — Description of Additional Supplementary Files [file 42004_2021_607_MOESM1_ESM.pdf]

Description of Additional Supplementary Files

File Name: Supplementary Data 1

Description: BI-BTCA CIF FILE

File Name: Supplementary Data 2

Description: BI-BTC CIF FILE
