# Supplementary material for: Bismuth organic frameworks exhibiting enhanced phosphorescence
Source: Commun Chem. 2021 Dec 2;4:167. doi: 10.1038/s42004-021-00607-x (PMC9814738; doi:10.1038/s42004-021-00607-x)
Supplement: Supplementary file 2 — Supplementary Information [file 42004_2021_607_MOESM2_ESM.pdf]

Supplementary Information

Bismuth organic frameworks exhibiting enhanced phosphorescence

Jin Young Koo[//], *Changmin Lee*[//], *Taiha Joo*[//], *Hee Cheul Choi*[*, //]

Department of Chemistry, Pohang University of Science and Technology (POSTECH), 77 Cheongam-ro, Namgu, Pohang, 37673, Korea

*To whom correspondence should be addressed. E-mail: choihc@postech.edu

Table of contents

Supplementary Materials and Methods S3

Supplementary Figures 1-15 S4-S18

Supplementary References S19

Supplementary Materials and Methods

Bismuth triiodide (BiI_3) (Sigma Aldrich, 99%), 1,2,4,5-Benzenetetracarboxylic acid (H_4BTCA) (Sigma Aldrich, 96.0%), 1,3,5-benzenetricarboxylic acid (H_3BTC) (Sigma Aldrich, 95.0%) N,N-dimethylformamide (Samchun, 99.0%), Toluene (ACS reagent, ≥99.5%), were purchased from commercial sources and used without further purification. All crystallization processes were conducted in a thermal oven (OF-11E, JEIOTECH, Korea). Solution UV-VIS spectra were recorded on a Shimadzu UV-VIS-NIR scanning spectrophotometer UV-2600. TGA spectra were obtained using a thermogravimetric analyzer (TGA, SCINCO model no.1000) from ambient temperature to ca. 800 ºC (heating rate of 10 ºC/min) under a continuous stream of nitrogen at a flow rate of 20 mL min^{-1}. The photoluminescence (PL) images and spectra were obtained using a fluorescence microscope (Olympus microscope) and spectrometer (SpectraPro, Prineton Instruments) equipped with a filter set (Exciter BP 330 – 380 nm; beam splitter 400 nm; Emitter LP 410 nm, Semrock). For CIE coordination index of its PL spectrum, the data were processed by Mathematica® 10 by using ChromaticityPlot on the CIE 1931 color space. The morphology of obtained crystals and the energy-dispersive X-ray spectra was characterized by High-Resolution FE-SEM-I (JSM 7401F). the sample is placed on a silicon wafer and coated with Pt to prevent charging of the non-conductive material for the high resolution data. The powder XRD patterns were recorded using a Bruker D8 Xray diffractometer. The simulated XRD patterns of **Bi-BTCA** were obtained using Mercury 3.0 programs.

Supplementary Figures

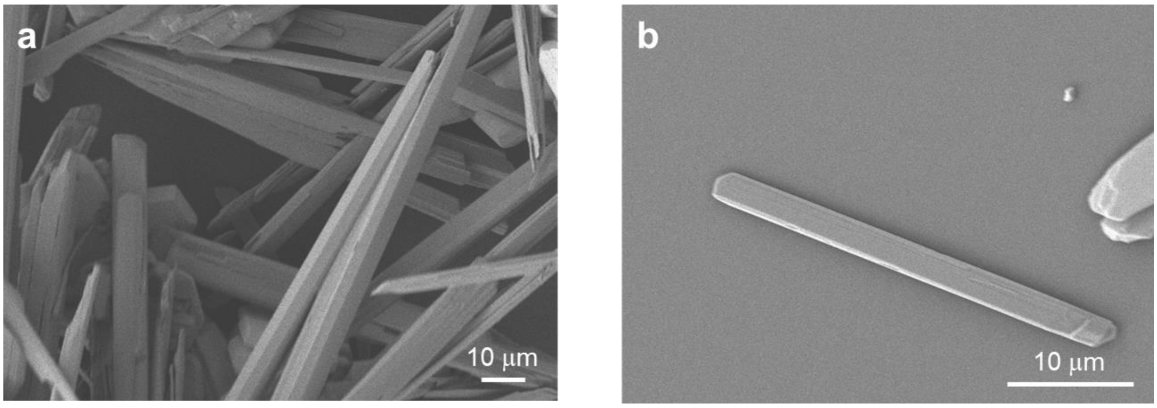

Supplementary Fig. 1 a) Low magnification and b) high magnification SEM images of colorless **Bi-BTCA** needle crystals.

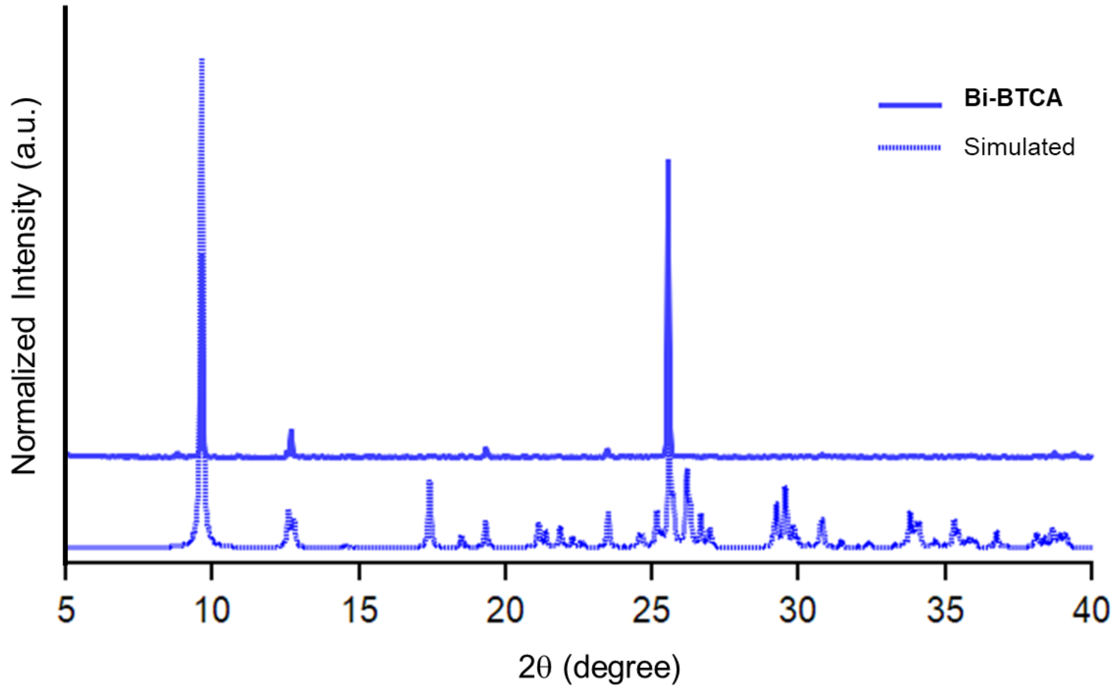

Supplementary Fig. 2 Comparison of experimental and simulated PXRD of **Bi-BTCA**.

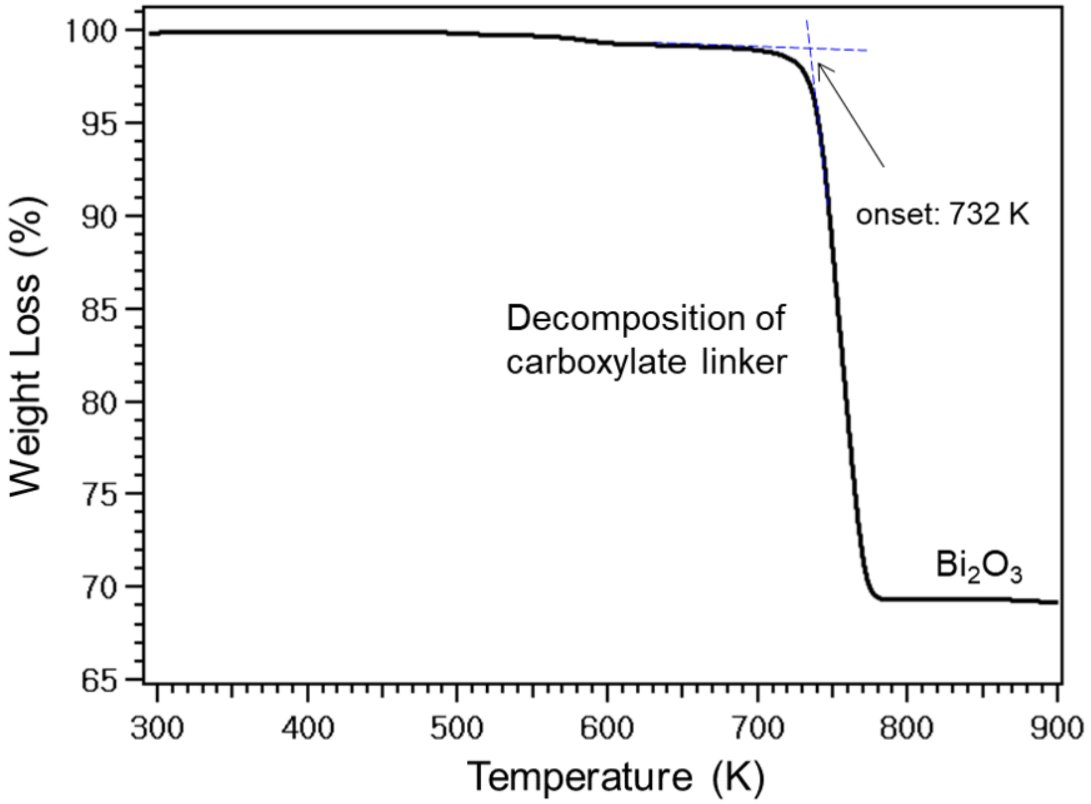

Supplementary Fig. 3 TGA curve of **Bi-BTCA** crystals. The first weight-loss stage of the network below 573 K was assigned to the removal of washing solvent. The decomposition of **Bi-BTCA** was observed at 731K (onset temperature). Samples were pretreated by washing with toluene and vacuum dried overnight.

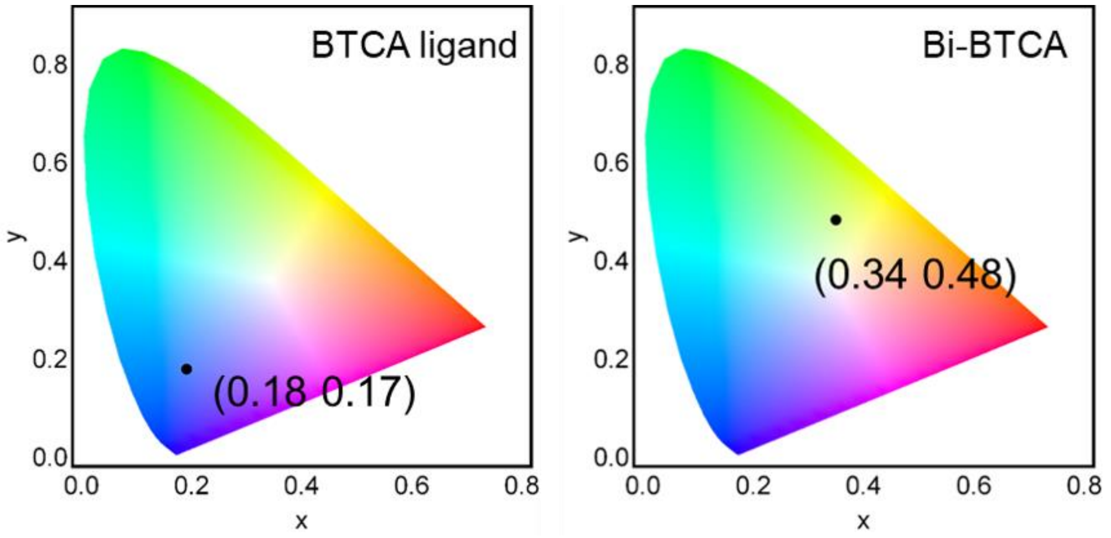

Supplementary Fig. 4 CIE x, y chromaticity diagram with coordinates corresponding to PL spectrum of BTCA ligand crystal and **Bi-BTCA** crystal. All samples emitted at the excitation wavelength of 356 nm.

Time-Resolved Photoluminescence

Homemade cavity-dumped Ti:Sapphire oscillator (760 nm, 20 fs, 380 kHz, 45 nJ) is used to measure the time-resolved photoluminescence (TRPL). Frequency doubling of the fundamental pulse using 300-μm-thick beta barium borate (β-BaB¬2O4, BBO) crystal makes 380 nm, 6 nJ pulse. The 380 nm pulse is used to excite the samples. Photoluminescence is collected into HPM100-07 detector after passing through the monochromator of 10 nm bandwidth. The time-correlated single photon counting (TCSPC) is measured using SPC130ENM module. The time resolution was 70 ps and the instrumental response function (IRF) is shown below.

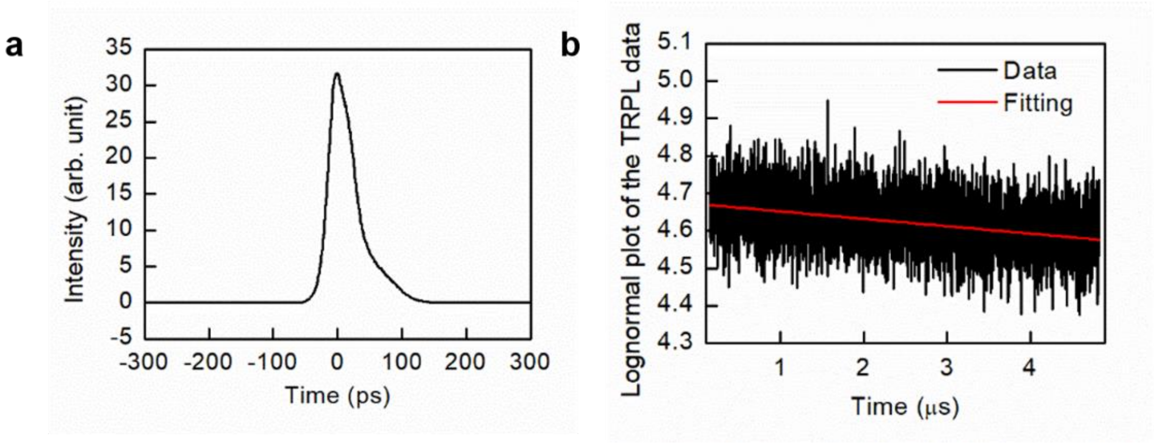

Supplementary Fig. 5 a) Instrumental response function of the TCSPC apparatus. The full width at the half maximum is fitted to be 70 ps assuming the Gaussian shape. b) The lognormal plot of the TRPL of **Bi-BTCA** with the longest time range of our instrument (5 μs). We estimated the longest time constant of **Bi-BTCA** from the slope of the lognormal plot assuming single decay component.

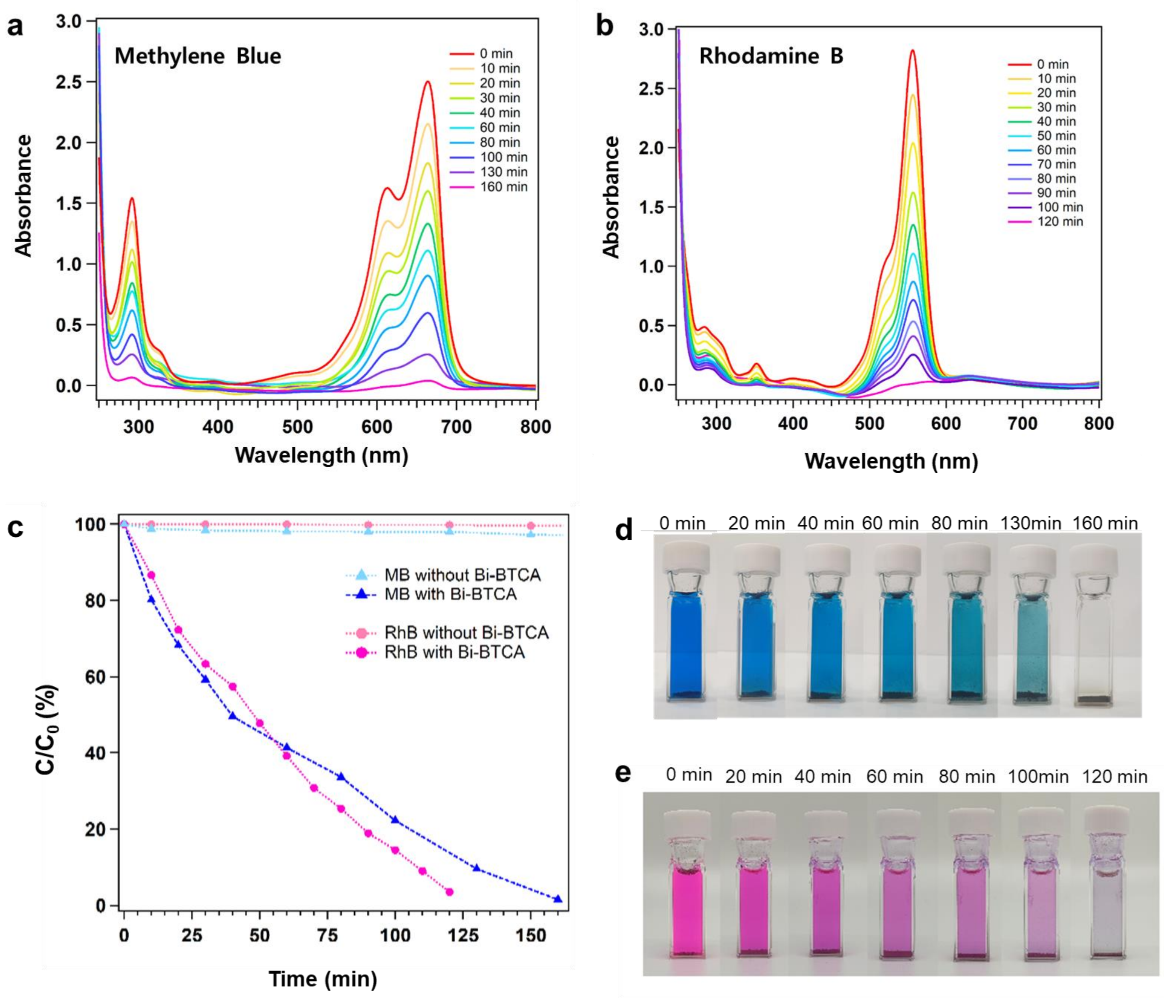

Supplementary Fig. 6 (a) and (b) Time-dependent UV-vis spectra of the MB solution or RhB solution of Bi-BTCA. (c) Photocatalytic activity of **Bi-BTCA** in dye degradation. (d) and (e) Photograph of the color change of MB and RhB solution, respectively.

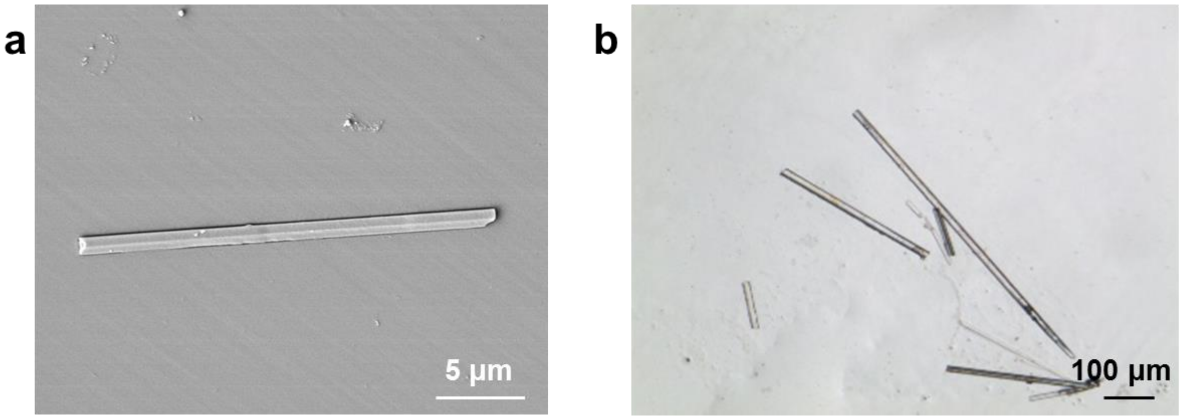

Supplementary Fig. 7 a) SEM and b) optical images of colorless **Bi-BTC** crystal.

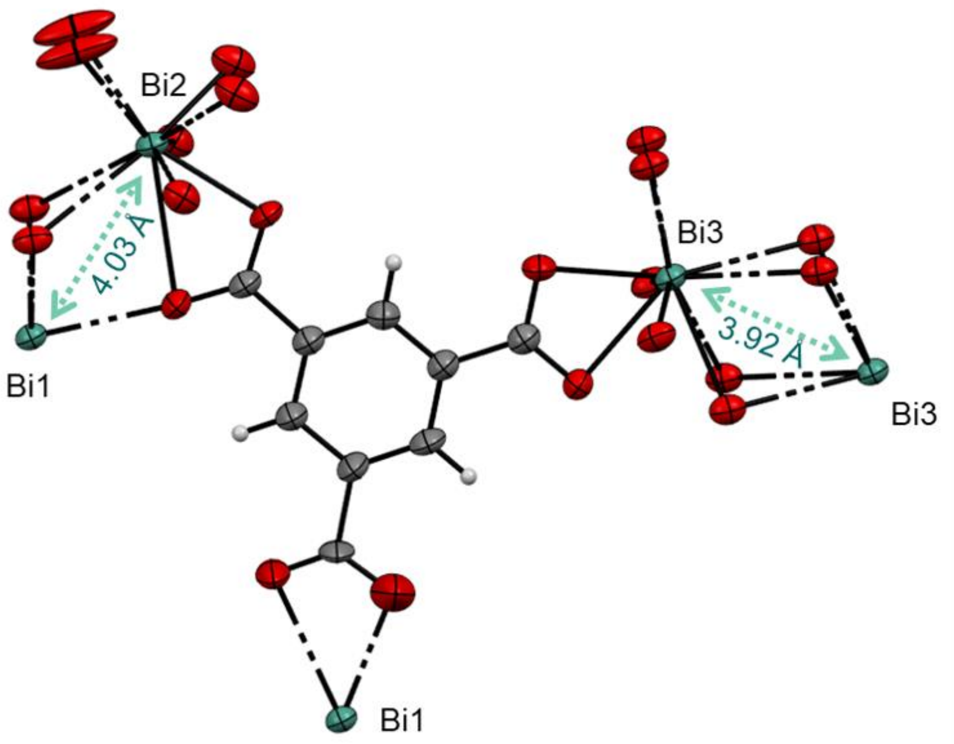

Supplementary Fig. 8 The ORTEP view of the asymmetric unit of the **Bi-BTC** with 50% thermal ellipsoids. H atoms are omitted for clarity.

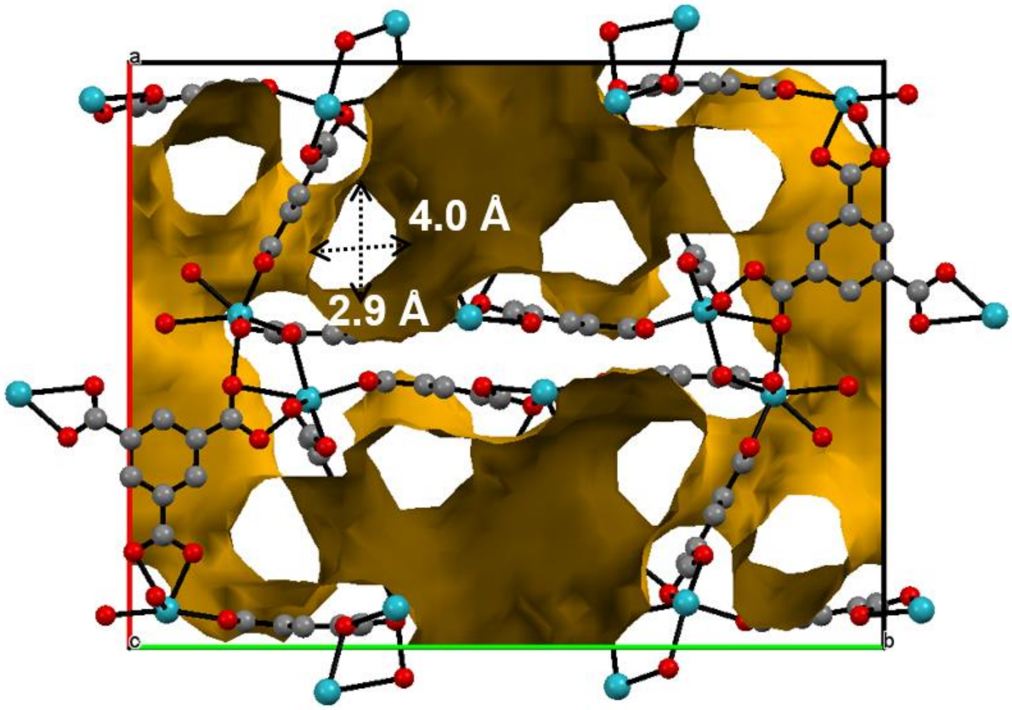

Supplementary Fig. 9 Pore morphologies and contact surface volumes (indicated by yellow surfaces). Calculations conduced in Mercury using a probe radius of 1.2 Å and grid spacing of 0.1 Å. Cyan, bismuth; gray, carbon; red, oxygen.

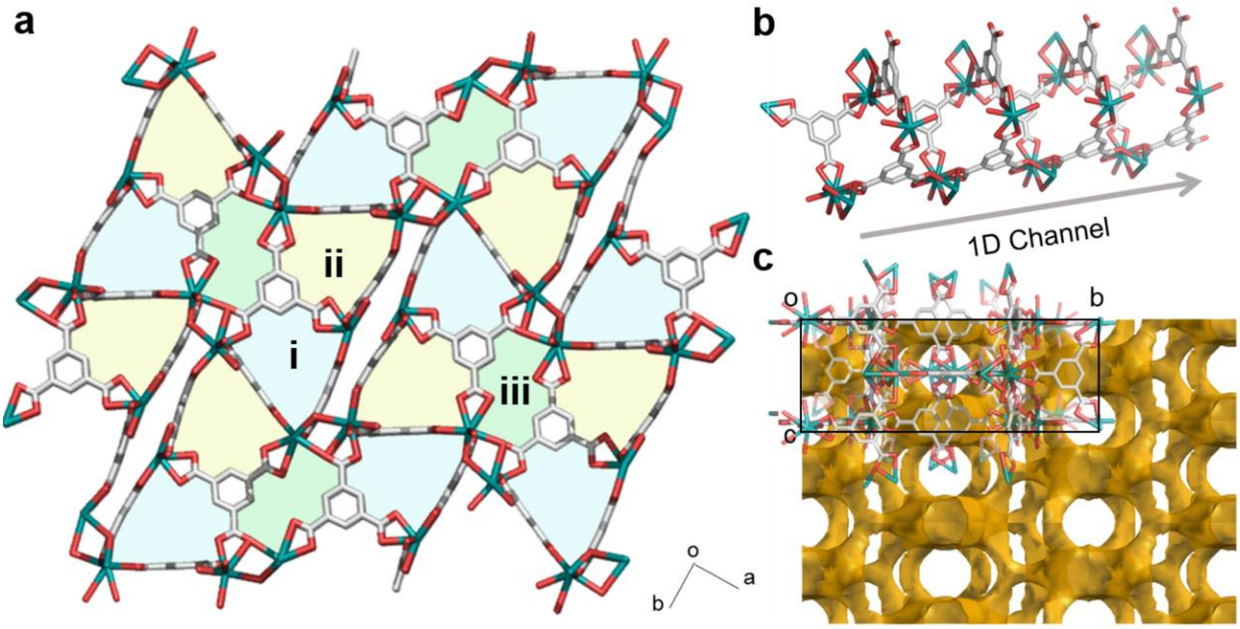

Supplementary Fig. 10 Crystal packing structure of **Bi-BTC**. a) the network has two trigonal pores (yellow, bule), and one independt rectangular pore (green). b)one dimensional channel along the *c* direction. c)simulated solvent accesible void space of **Bi-BTC** calculated on Mercury 3.0. Although hydrogen atoms are omitted here for clarity, they were considered in the calculation of the accessbile volumes. Green, bismuth; gray, carbon; red, oxygen.

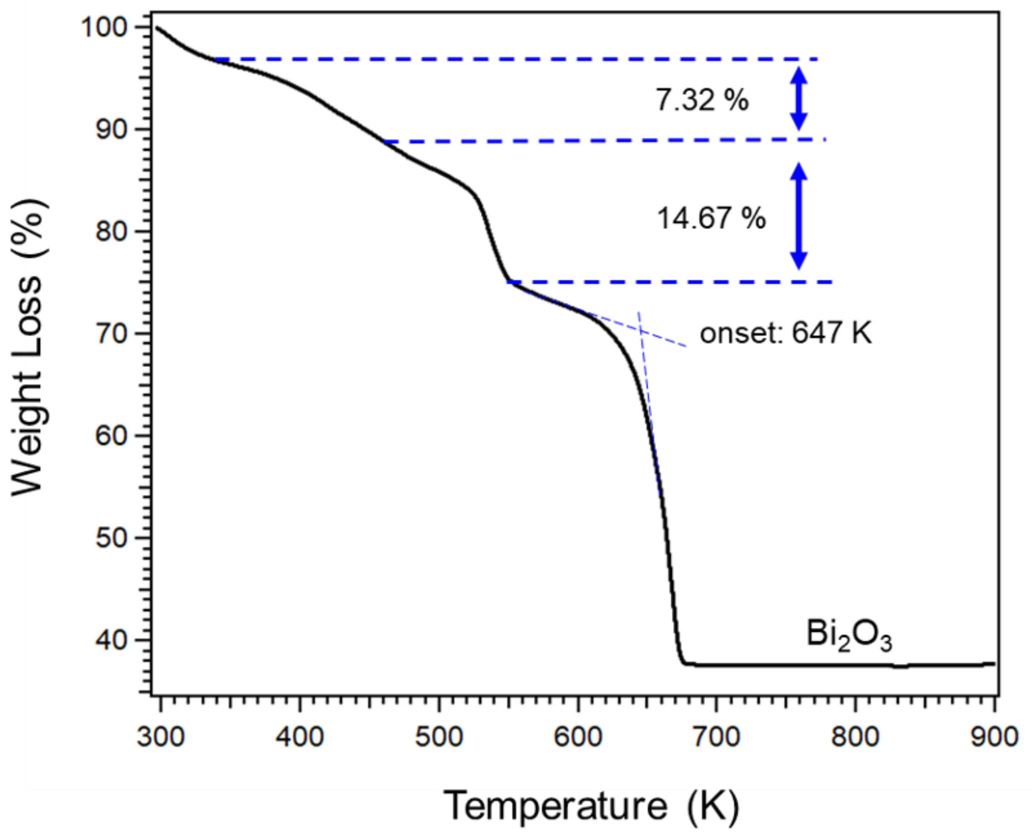

Supplementary Fig. 11 TGA curve of **Bi-BTC** crystals. The first weight-loss stage of the network below 339 K was assigned to the removal of toluene molecules. During the second stage, between 339 K and 452 K, the network underwent slow decomposition, around 14.67%, releasing N,N-dimethylformamide (DMF). The third stage of weight loss links to the **Bi-BTC** decomposition.

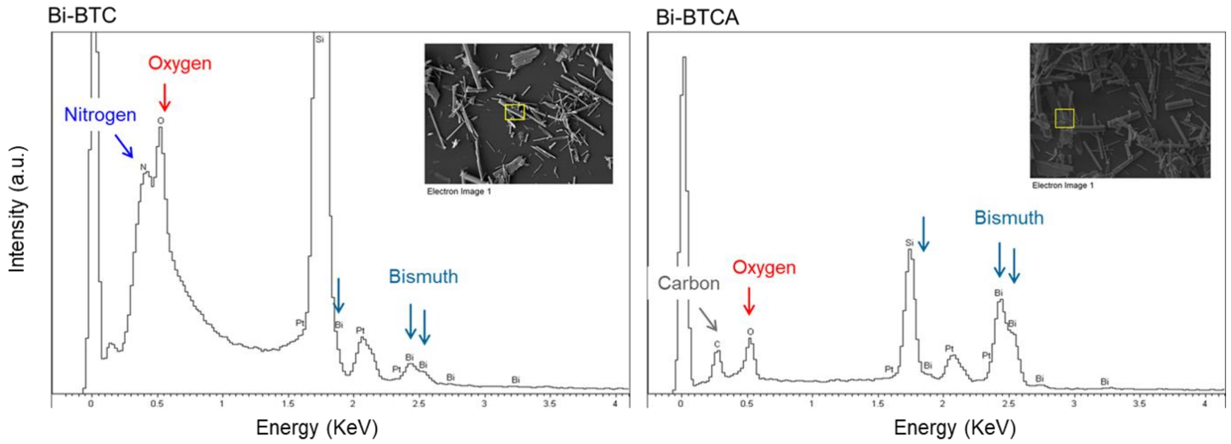

Supplementary Fig. 12 Energy-dispersive X-ray Spectrum of **Bi-BTC** and **Bi-BTCA**.

a

	Anthracene	Bi-BTC	Bi-BTCA
Emission Area	504969.0535	5284.418987	75737.11771
Extincted Photon Power	0.16	0.11	0.09
Quantum Yield	0.64	0.009741812	0.1706481

b

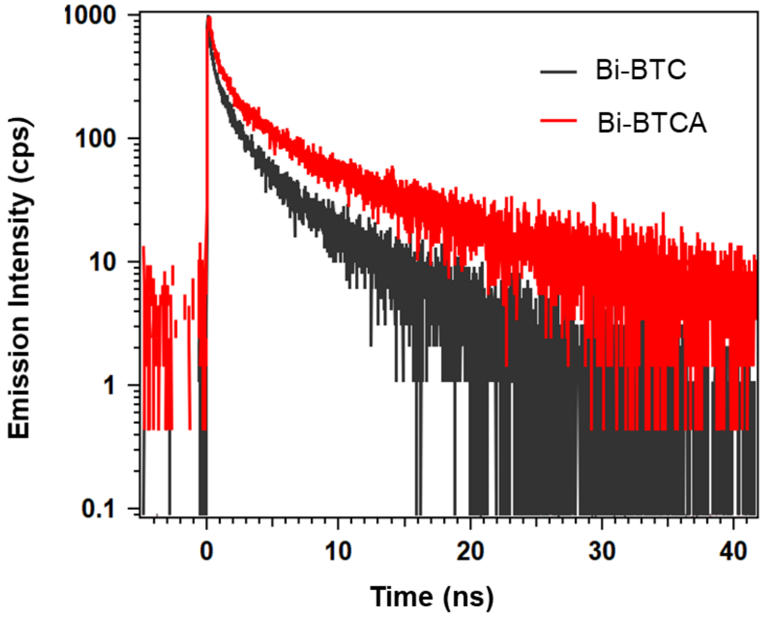

Supplementary Fig. 13 a) Photoluminescence quantum yields obtained in single crystal b) TRPL of **Bi-BTCA** (red) and **Bi-BTC** (grey) crystal detected at 500 nm after photoexcitation at 380 nm. Time resolution was 75 ps.

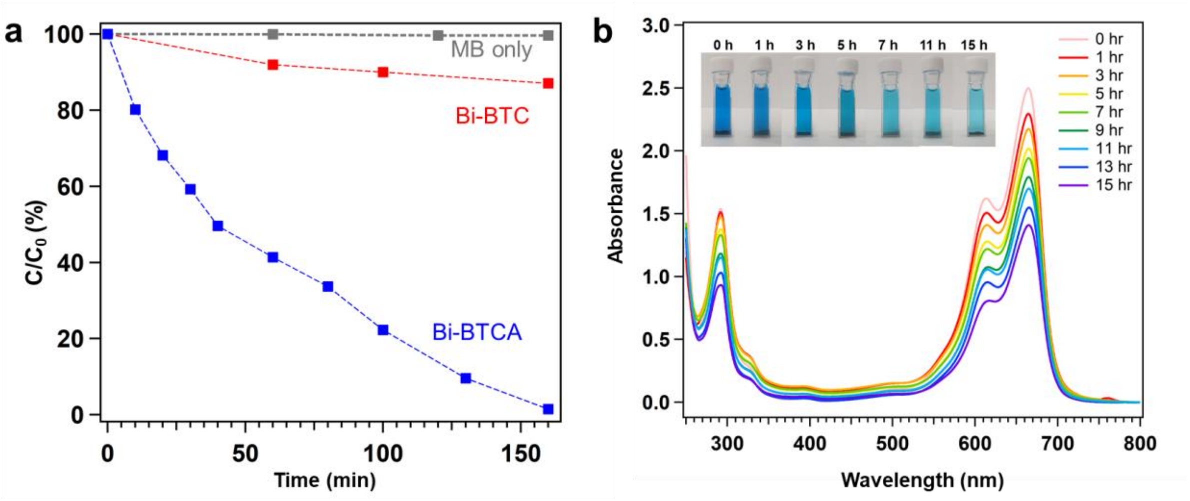

Supplementary Fig. 14 (a) Photocatalytic activity BiOFs in MB degradation. **Bi-BTCA** (blue), **Bi-BTC** (red) (b) Time-dependent UV-vis spectra of the MB solution of **Bi-BTC**. Inset: photograph of the color change of the MB solution

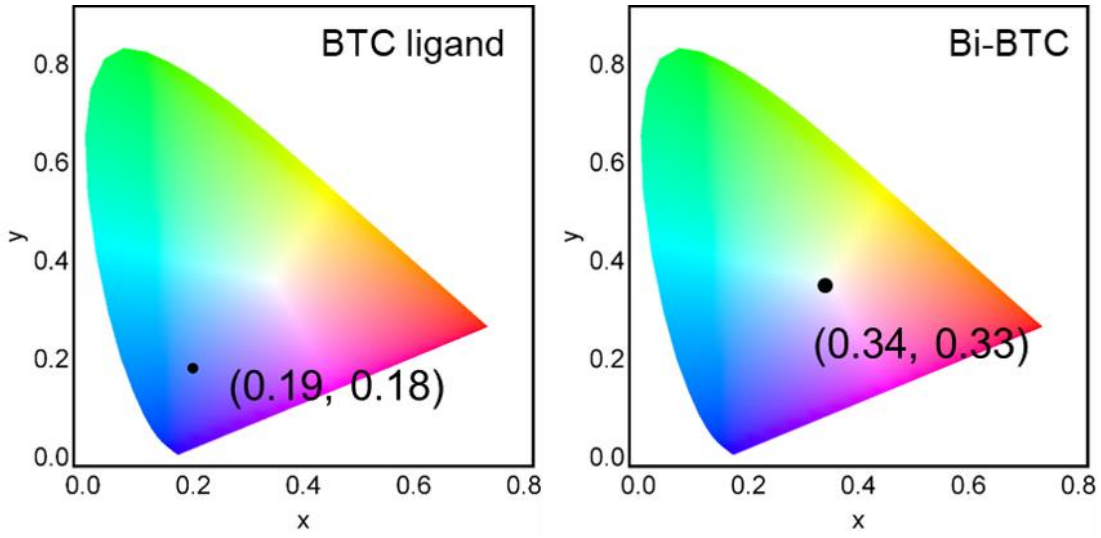

Supplementary Fig. 15 CIE x, y chromaticity diagram with coordinates corresponding to PL spectrum of a) BTC ligand and **Bi-BTC** crystal. All samples emitted at the excitation wavelength of 356 nm.

Supplementary References

S1. A. J. Arvai and C. Nielsen, ADSC Quantum-210 ADX Program, Area Detector System Corporation; Poway, CA, USA, 1983.

S2. Z. Otwinowski, W. Minor, in Methods in Enzymology, ed. C. W. Carter, Jr. and R. M. Sweet, Academic Press, New York, 1997, vol. 276, part A, pp. 307.

S3. PLATON program: A. L. Spek, Acta Cryst., 2009, D65, 148

S4. SHELX program: G. M. Sheldrick, Acta Cryst., 2008, A64, 112.
